# Enzyme-Assisted Fucoidan Extraction from Brown Macroalgae *Fucus distichus* subsp. *evanescens* and *Saccharina latissima*

**DOI:** 10.3390/md18060296

**Published:** 2020-06-02

**Authors:** Thuan Thi Nguyen, Maria Dalgaard Mikkelsen, Vy Ha Nguyen Tran, Vo Thi Dieu Trang, Nanna Rhein-Knudsen, Jesper Holck, Anton B. Rasin, Hang Thi Thuy Cao, Tran Thi Thanh Van, Anne S. Meyer

**Affiliations:** 1 Protein Chemistry and Enzyme Technology Section, DTU Bioengineering, Department of Biotechnology and Biomedicine, Technical University of Denmark, Building 221, 2800 Kongens Lyngby, Denmark; thuthi@dtu.dk (T.T.N.); vyha@dtu.dk (V.H.N.T.); tvtd@dtu.dk (V.T.D.T.); nark@dtu.dk (N.R.-K.); jesho@dtu.dk (J.H.); asme@dtu.dk (A.S.M.); 2 NhaTrang Institute of Technology Research and Application, Vietnam Academy of Science and Technology, 02 Hung Vuong Street, Nhatrang 650000, Vietnam; caohang.nitra@gmail.com (H.T.T.C.); tranthanhvan@nitra.vast.vn (T.T.T.V.); 3 G.B. Elyakov Pacific Institute of Bioorganic Chemistry, Far-Eastern Branch of the Russian Academy of Sciences, 159, Prospect 100-let Vladivostoku, Vladivostok 690022, Russia; Abrus__54@mail.ru

**Keywords:** fucose-containing sulfated polysaccharides, *Fucus evanescens*, enzymatic extraction, chemical extraction, cellulase, alginate lyase

## Abstract

Fucoidans from brown macroalgae (brown seaweeds) have different structures and many interesting bioactivities. Fucoidans are classically extracted from brown seaweeds by hot acidic extraction. Here, we report a new targeted enzyme-assisted methodology for fucoidan extraction from brown seaweeds. This enzyme-assisted extraction protocol involves a one-step combined use of a commercial cellulase preparation (Cellic^®^CTec2) and an alginate lyase from *Sphingomonas* sp. (SALy), reaction at pH 6.0, 40 °C, removal of non-fucoidan polysaccharides by Ca^2+^ precipitation, and ethanol-precipitation of crude fucoidan. The workability of this method is demonstrated for fucoidan extraction from *Fucus distichus* subsp. *evanescens* (basionym *Fucus evanescens*) and *Saccharina latissima* as compared with mild acidic extraction. The crude fucoidans resulting directly from the enzyme-assisted method contained considerable amounts of low molecular weight alginate, but this residual alginate was effectively removed by an additional ion-exchange chromatographic step to yield pure fucoidans (as confirmed by ^1^H NMR). The fucoidan yields that were obtained by the enzymatic method were comparable to the chemically extracted yields for both *F. evanescens* and *S. latissima*, but the molecular sizes of the fucoidans were significantly larger with enzyme-assisted extraction. The molecular weight distribution of the fucoidan fractions was 400 to 800 kDa for *F. evanescens* and 300 to 800 kDa for *S. latissima*, whereas the molecular weights of the corresponding chemically extracted fucoidans from these seaweeds were 10–100 kDa and 50–100 kDa, respectively. Enzyme-assisted extraction represents a new gentle strategy for fucoidan extraction and it provides new opportunities for obtaining high yields of native fucoidan structures from brown macroalgae.

## 1. Introduction

Fucoidans are a group of sulfated polysaccharides mainly found in the cell walls of brown seaweeds. Polysaccharides are intensively studied due to their useful bioactivities, such as antioxidant [1], antitumor [2], neuroprotective [3], anti-inflammatory, and anticoagulant [4]. Fucoidan content and monosaccharide composition depend on many factors, such as seaweed species, environmental conditions, collecting time, and methods used for polysaccharide extraction and purification [5,6,7]. Fucoidans are heteropolymers that are primarily composed of fucose, but may include other sugars, i.e. glucose, galactose, xylose, mannose, and uronic acids in various proportions. Based on the sugar composition, fucoidans from brown seaweeds were previously reported as sulfated fucans, mainly containing fucose and sulfate groups, galactofucans mainly consisting of sulfated fucose and galactose in different ratios, and urono-fucoidans with different monosaccharide units, less sulfate, and a large amount of uronic acid [8]. Fucoidans differ in their backbone structure and branching patterns and in presence and numbers of functional groups, such as sulfates and acetylations. The backbone consists most commonly of α-(1–3)-l-fucopyranose units or alternating α-(1–3)- and α-(1–4)-linked l-fucopyranose units with a sulfate group at C2 or C4 [9]. In general, the backbone of fucoidans from *Fucus distichus* subsp. *evanescens* (a species that we in this report refer to as *F. evanescens*) is made up of alternating 2-sulfated 1,3- and 1,4-linked α-l-fucose residues [10,11], although on some of the fucose residues sulfate groups may be found at both C2 and C4 [12]. Fucoidans from *Saccharina latissima* are very diverse. Four partial structures of fucoidans from *S. latissima* have been reported: fucan sulfate, fucogalactan, fucoglucuronomannan, and fucoglucuronan [13].

The bioactivities of fucoidans from different species of brown seaweeds are closely related to their monosaccharide composition, molecular weight, and fine structure [14,15,16,17,18]. The cell wall of brown seaweeds is a complex matrix of compounds, primarily consisting of the polysaccharides alginate, fucoidans, cellulose, and hemicellulose, as well as proteins, polyphenols, and other components [19].

The main goal when extracting fucoidans is to isolate the fucoidans as intact as possible while eliminating interfering molecules. Traditional fucoidan extraction techniques (acidic, hot water, organic solvents) are based on the solubility of cell wall polysaccharides under various conditions. But these methods are time consuming, toxic, have low efficiency and may affect the fucoidan fine structure, which potentially could have a detrimental effect on fucoidan bioactivity. Novel green fucoidan extraction techniques have been developed to overcome these disadvantages, including supercritical water extraction [20], microwave-assisted extraction [21], ultrasound-assisted extraction [22], and enzyme-assisted extraction [23]. Enzyme-assisted fucoidan extraction should preferably be based on selective removal of all non-fucoidan polysaccharides from the cell wall matrix and also target depolymerization of the storage polysaccharide laminarin, and leave the sulfated fucoidan intact. However, until now, the reported enzyme-assisted extraction methods for sulfated fucoidan polysaccharides have involved use of various mixed commercial enzyme preparations, such as carbohydrase mixtures (Viscozyme, Celluclast, Amyloglucosidase), known to attack a wide range of plant polysaccharides (even including starch, which is not present in brown seaweeds) and broadly acting proteases (Flavourzyme, Alcalase) [23,24,25]. Hence, these enzyme mixtures do not selectively target alginate, which is a particularly abundant polysaccharide in the cell walls of brown macroalgae. Alginate is particularly important to remove enzymatically to release fucoidan, because fucoidans are believed to be involved in the cross-linkage of alginate and cellulose [26]. Therefore, it is important to judiciously select specific substrate-targeted enzymes, i.e. alginate lyase and cellulases, which specifically catalyze the degradation of the fucoidan cross-linking polysaccharides in order to selectively extract fucoidans from the cross-linked network of brown algae cell walls in a controlled and gentle way by enzymes. This concept, i.e. the combined use of alginate lyase and cellulases, is the foundation of the new, targeted enzyme-assisted method that we report here. In a previous study, cell wall treatment with alginate lyase only released a minor fraction of the fucose-containing sulfated polysaccharides [27].

In this study, we report the comparative isolation of fucoidans from two different brown seaweeds, *F. evanescens* and *S. latissima*, by acid extraction and enzymatic extraction while using Cellic^®^CTec2 from Novozymes and the alginate lyase SALy from *Sphingomonas sp*. [28]. The reason for choosing these species for study is that they are both available in the Northern hemisphere zone, and *S. latissima* is moreover commercially cultivated by Nordic companies, which is why it is particularly useful to assess fucoidan extraction from this species for value addition. *F. evanescens* is collected for investigating potential commercial use of the fucoidan and known as a species that is quite rich in fucoidan. In addition, since *S. latissima* is of the order Kelp and *F. evanescens* belongs to the Fucales, these two species would serve to demonstrate the workability of the enzyme-assisted extraction method for two significantly different, relevant types of brown algae to provide a robust case. The fucose extraction yield, monosaccharide composition, and molecular size of crude fucoidans from these two seaweeds were analyzed. Furthermore, the crude fucoidans from *F*. *evanescens* and *S. latissima* were fractionated by anion-exchange chromatography into three fractions. We also studied the structural and molecular properties of these fucoidan fractions.

## 2. Results

### 2.1. Monosaccharide Composition of the Brown Seaweeds F. evanescens and S. latissima

The monosaccharide composition of *F. evanescens* and *S. latissima* was analyzed in order to evaluate the effect of using enzymes to extract fucoidans from brown seaweeds (Table 1).

The predicted monosaccharides were found in different proportions in the seaweeds. The neutral monosaccharides found were mannitol, fucose, rhamnose, galactose, glucose, xylose and mannose, and uronic acids included mannuronic acid (ManA), guluronic acid (GuluA), and glucuronic acid (GluA). Fucose is the major component of fucoidans, while glucose is a component of laminarin or cellulose, and ManA and GuluA are components of alginic acid (alginate). Hence, the data presented in Table 1 are in agreement with fucoidans, laminarin, cellulose, and alginate, being the main polysaccharides present in *F. evanescens* and *S. latissima* constituting about 60% and 68%, respectively, of the dry matter (sum of data in Table 1). The content of fucose in *F. evanescens* and *S. latissima* differs, and as expected, *F. evanescens* contained the highest levels: Hence, in *F. evanescens*, fucose comprised 8.7% of the dry weight, which is considerably higher than the 4.7% found in *S. latissima* (Table 1). Furthermore, fucoidans extracted from these two species of seaweeds have previously been reported to have different chemical composition and structure [10,13,29].

The content of alginate in *F. evanescens* and *S. latissima* was very high at 37.1% and 45.4% of the dry weight in the two seaweeds, respectively, (calculated as the sum of guluronic acid and mannuronic acid levels in each seaweed, Table 1), which was anticipated [30]. Lower amounts of other neutral sugars found in fucoidan were also present in *F. evanescens* and *S. latissima*: galactose (1.5% and 0.5%), xylose (0.8% and 0.4%), and mannose (0.9% and 1%). Additionally, some glucuronic acid, which is also a component of fucoidans, such as the fucoglucuronomannan and fucoglucuronan fucoidans reported in *S. latissima* [13], was also present in lower amounts in *F. evanescens* (1.2%) and *S. latissima* (1.9%) (Table 1).

In a previous study from our lab, we found that the alginate lyase SALy and the commercial cellulase enzyme preparation Cellic^®^CTec2 could be combined to release glucose from the biomass of the brown seaweed *Laminaria digitata* [28]. In the same study, it was suggested that the fucoidans were released from the cell wall unharmed, although this possibility was not tested further; in another study, Cellic^®^CTec2 was shown to be able to catalyze the degradation of laminarin from seaweeds [31].

### 2.2. Enzyme-Assisted Fucoidan Extraction from F. evanescens and S. latissima

In the first step of the enzyme-assisted fucoidan extraction method, SALy and Cellic^®^CTec2 were combined to trigger fucoidan extraction from *F. evanescens* and *S. latissima*. A mild chemical acidic extraction was used for comparison in order to evaluate the effectiveness of the enzyme-assisted method.

#### 2.2.1. Yields of Crude Fucoidans Extracted from *F. evanescens* and *S. latissima*

The amount of fucose was used to determine the extraction efficiency of fucoidans by comparing the ratio of total fucose in crude fucoidans and the content in the starting material. The chemical composition of crude fucoidans obtained by the chemical and enzymatic extraction methods from *F. evanescens* and *S. latissima* was analyzed by high performance anion chromatography with pulsed amperometric detection (HPAEC-PAD) (Table 2).

The crude fucoidan extracts from *F. evanescens* and *S. latissima* were mainly composed of four types of neutral sugars, fucose, galactose, glucose, and xylose, and a small amount of mannose and rhamnose and we also consider the low levels of the uronic acid glucuronic acid to be a part of the fucoidan (Table 2). A major part of the glucose in the original seaweed materials (Table 1) is presumed to originate from cellulose and laminarin, and the crude fucoidan data show that both are effectively degraded because glucose is removed by the enzymatic treatment. Thus, glucose levels were considerably lower in the enzyme-assisted extracts when compared to the chemical extractions, at 0.7% as compared to 6.2% in *F. evanescens* and 1.6% as compared to 57.7% in *S. latissima* (Table 2); this result was in complete accord with the expected effect of the Cellic^®^CTec2 acting on both cellulose and laminarin [31]. We interpret the presence of GuluA and ManA as being indicative of alginate contamination. Indeed, the relative levels of both GuluA and ManA were considerably higher for the enzymatic extraction when compared to the chemical extraction, namely 58.4% ManA as compared to 13.1% in *F. evanescens* and 59.6 % ManA when compared to 1.0% in *S. latissima* (Table 2). This difference led to the *relative* fucose contents being higher with the chemical extraction than with the enzyme-assisted extraction, at 60.9% as compared to 24.8% in *F. evanescens* and 31.2% when compared to 12.6% in *S. latissima* (Table 2). Yet, the total yields of fucoidan were comparable between the two methods being 29% with both methods for *S. latissima* and 40% and 43% for *F. evanescens* (Table 2).

The sulfation degree tended to be slightly higher in the enzymatically-assisted extracted fucoidans than in the chemically extracted at 2.1% as compared to 1.9% in the crude *F. evanescens* fucoidan and 2.5% compared to 2.1% in the *S. latissima* fucoidan extracts (Table 2). The finding that crude fucoidans from *F. evanescens* and *S. latissima* were highly sulfated polysaccharides is in complete accordance with what has been reported previously [10,13].

#### 2.2.2. Yields and Chemical Compositions of Fucoidan IEX Fractions of *F. evanescens* and *S. latissima*

**Figure 1:**
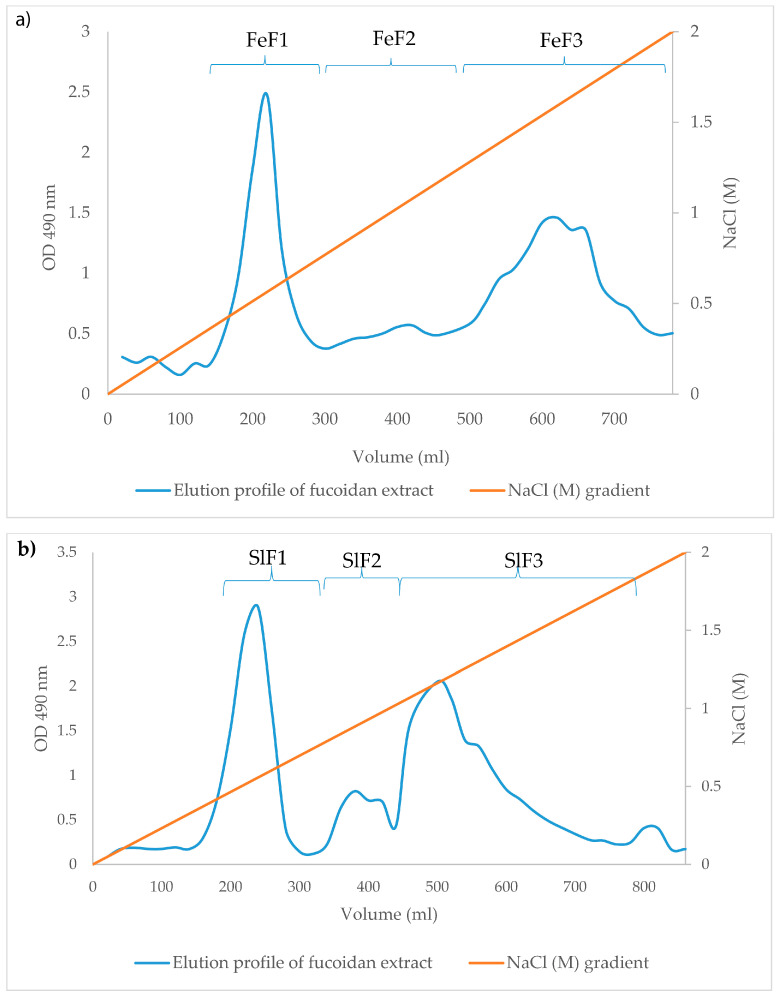
The elution profile of ion-exchange chromatography (IEX) fractionated fucoidan. (**a**) Elution profile for the *F. evanescens* chromatography purificatonand. (**b**) Elution profile for the *S. latissima* fucoidan during IEX. Based on the elution profile, three extracts FeF1–F3 and SlF1–F3 were collected from the *F. evanescens* and *S. latissima* purifications, respectively.

Crude fucoidans from the enzyme-assisted purifications were further purified and separated by ion-exchange chromatography (IEX). This method allows for different populations of fucoidans to be separated according to differences in size and charge. The polysaccharides were eluted depending on negative charge intensity by increasing salt concentrations. Based on the total carbohydrate content, determined by the phenol-sulfuric acid method [32] of each eluate, the eluates of *F. evanescens* and *S. latissima* were combined into three different fractions FeF1–F3 and SlF1–F3, respectively (Figure 1). The yield and composition of these fucoidan fractions were different with regards to monosaccharide distribution and sulfate content (Table 3).

FeF1 consisted of fucose (34%), galactose, glucose, xylose, mannose, traces of rhamnose, and a high level of ManA (32.2%) (Table 3). Most uronic acids from alginate contamination eluted in FeF1. Fucose was the major component found in FeF2 and FeF3 at 74.7% and 87.8%, while the galactose content was 15.4% and 9.0% in these two fractions, respectively. Furthermore, the amount of glucose was very low, 1.4% and 0.3% in FeF2 and FeF3 (Table 3), respectively, suggesting that laminarin and cellulose had been successfully removed. Likewise, because alginate had been eluted in FeF1, its content was very low in FeF2 and FeF3 at 2.4% and 0%, respectively. The content of sulfate was also high with 34.8% in FeF2 and 38.7% in FeF3.

Three fucoidan fractions SlF1–F3 were obtained based on the elution profile of extracts from *S. latissima* (Figure 1b) (Table 3). Fraction SlF1 almost exclusively consisted of ManA (82.4%) and the content of neutral sugars was low and included only 5.4% fucose. Fractions SlF2 and SlF3 mainly consisted, respectively, of fucose at 64.7% and 63.3% and galactose at 12.2% and 26.9%. Alginic acid content was low with a slightly higher concentration in SlF2 (13.8%) in comparison to SlF3 (3.6%), while the glucose content was very low in both SlF2 (0.6%) and SlF3 (0.4%). Sulfate content was also high in SlF2 (35.6%) and SlF3 (46.4%) when compared to only 6.6% in SlF1, which was consistent with the amount of fucoidan in the samples.

The current analysis indicates that FeF2, FeF3, SlF2, and SlF3 can be considered pure fucoidans due to high levels of fucose, galactose, and sulfates and low amounts of glucose and alginate.

### 2.3. Size Exclusion Chromatography (SEC) Analysis

#### 2.3.1. SEC of Crude Fucoidans

**Figure 2:**
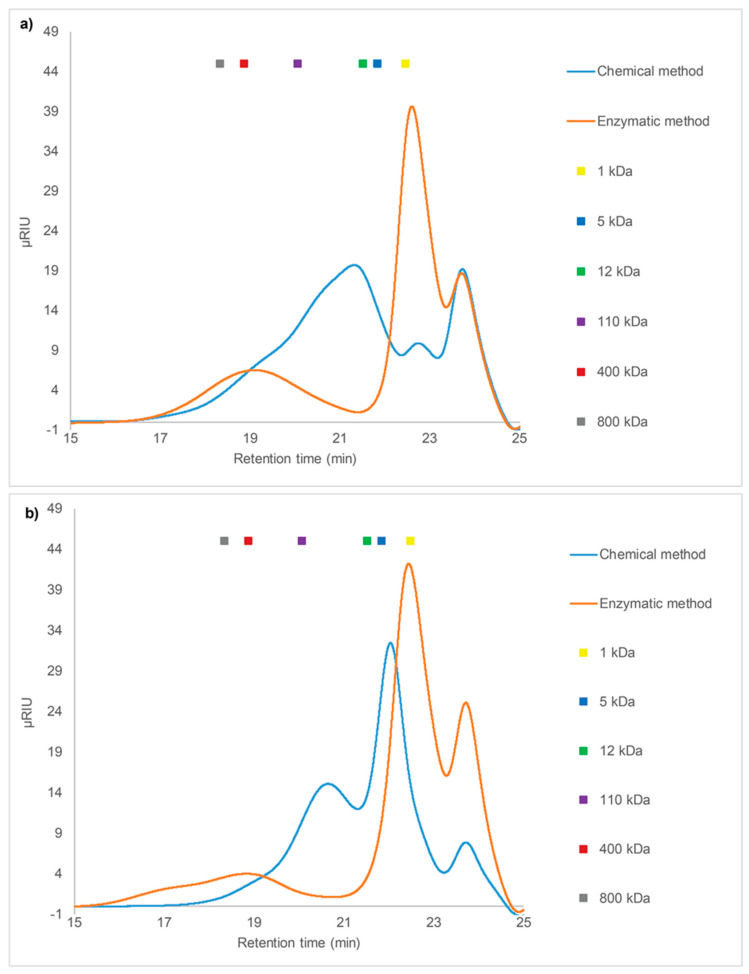
SEC chromatogram of crude fucoidans from chemical and enzyme-assisted purification. (**a**) *F. evanescens* and (**b**) *S. latissima*. Pullulan was used as standard.

Extraction procedures can affect the molecular weight of fucoidans and molecular weight is an important factor that can influence fucoidan bioactivity [6]. The enzyme-assisted extraction procedure was expected to result in intact fucoidans with larger molecular weight than that obtained by chemical extraction. Thus, in this study, the mass distribution of enzymatic and chemically extracted crude fucoidans from *F. evanescens* and *S. latissima* were compared. The molecular weight of crude fucoidans normally ranges from 21 to 1600 kDa [33]. All crude extracts showed heterogeneous molecular weight profiles based on High Performance Size Exclusion Chromatography (HP-SEC) analysis (Figure 2). The enzymatic extracts from *F. evanescens* consisted of two different populations of polymers, one with a molecular weight less than 5 kDa and a second with a broad distribution with molecular weights from approximately 50 kDa to more than 800 kDa and averaging 100–800 kDa. A more heterogeneous distribution in the range of less than 1 kDa to approximately 800 kDa was observed for the chemical extract, and an average molecular weight of 10–100 kDa, i.e. considerably lower than for the enzyme-assisted purification (Figure 2a), and the very low molecular weight species (<21 kDa) may, in a strict definition, not be defined as fucoidan molecules. The same pattern was observed for the enzymatic extract of *S. latissima*, which gave two different populations, one with a molecular mass less than 10 kDa and one from ~100 kDa to over 800 kDa, with the latter population being larger and more broadly distributed compared to *F. evanescens* (Figure 2b). The chemical extract of *S. latissima* was better defined than the *F. evanescens* extract, with two defined populations, one of less than 5 kDa and the second with approximately 50−100 kDa (Figure 2b).

The difference in molecular weights between fucoidans from *F. evanescens* and *S. latissima* shows the size diversity of native fucoidans in brown seaweeds. The HP-SEC results thus showed that the enzyme-assisted technique resulted in the extraction of fucoidans with a generally higher molecular weight as compared to the chemical technique for these two seaweed species.

#### 2.3.2. SEC of Fucoidan Fractions from *F. evanescens* and *S. latissima*

The IEX fractionated extracts were also analyzed by HP-SEC and they showed different molecular weight distributions of the different fucoidan fractions from *F. evanescens* and *S. latissima*.

**Figure 3:**
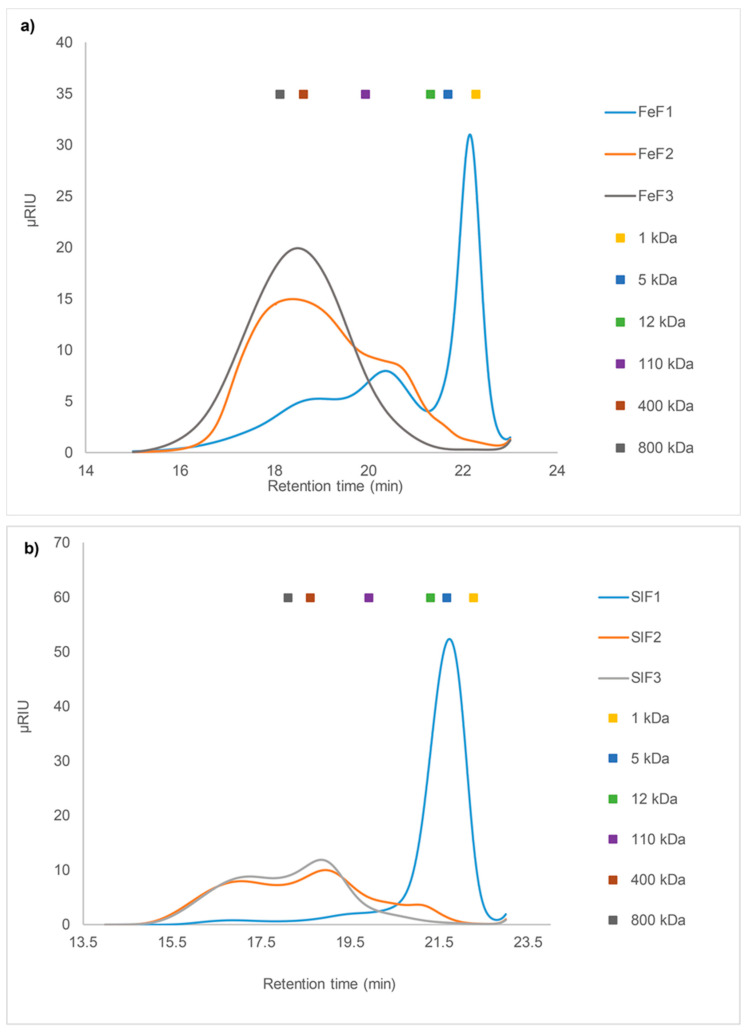
SEC chromatogram of fucoidan fractions after IEX purification. (**a**) *F. evanescens*: the FeF1 fraction contains LMW compounds of around 2 kDa and a smaller proportion of HMW compounds between ~100–500 kDa, while FeF2 and FeF3 contain primarily HMW compounds ranging from ~400 kDa to ~800 kDa; (**b**) *S. latissima*: the SlF1 fraction contains almost exclusively LMW compounds of around 5 kDa, while SlF2 and SlF3 contain more HMW compounds ranging from ~300 kDa to over 800 kDa. Pullulan was used as standard.

The *F. evanescens* FeF1 consisted of poly- and oligosaccharides with a very wide range of molecular weight distribution and three main peaks at 2–3 kDa, 30–40 kDa, and ~400 kDa (Figure 3a). This suggests the presence of many different components, which was also evident in the monosaccharide composition analysis. A high content of ManA was observed for FeF1, therefore the peak at 2–3 kDa is suggested to be oligo-alginate and poly-ManA contaminants from the SALy catalyzed alginate degradation. FeF2 contained a broad molecular weight distribution from approximately 30 kDa to over 800 kDa, with the largest peak around 400–800 kDa and a small shoulder around 30–40 kDa, as was observed in FeF1. The shoulder might represent smaller sized fucoidans, since FeF2 only contains very low amounts of alginate (2.4%). FeF3 contained one homogeneous peak around 400–500 kDa (Figure 3a), and monosaccharide analysis of FeF3 (Table 3) confirmed the presence of highly pure fucoidans.

*S. latissima* fractions showed a different molecular weight distribution than that observed for *F. evanescens* and the size distribution of the populations of fucoidan molecules seemed more complex than those from *F. evanescens* (Figure 3b). The major component of SlF1 was a highly homogeneous peak of less than 5 kDa, and monosaccharide analysis showed the main carbohydrate content to be ManA (Table 3). SlF2 and SlF3 had similar SEC profiles, with a molecular weight distribution in the range of approximately 300 kDa to over 800 kDa (Figure 3b). In addition, SlF2 had a small shoulder of around 10 kDa, likely due to the presence of minor alginate impurities and confirmed by the monosaccharide composition (13.8%). The monosaccharide composition of SlF2 and SlF3 implied pure fucoidans (Table 3), although the mass distribution of the fractions appeared to be rather heterogeneous. This could indicate that the fucoidans from *S. latissima* have very complex structures.

### 2.4. ^1^H NMR Spectrum of Fucoidan Fractions from *F. evanescens* and *S. latissima*

**Figure 4:**
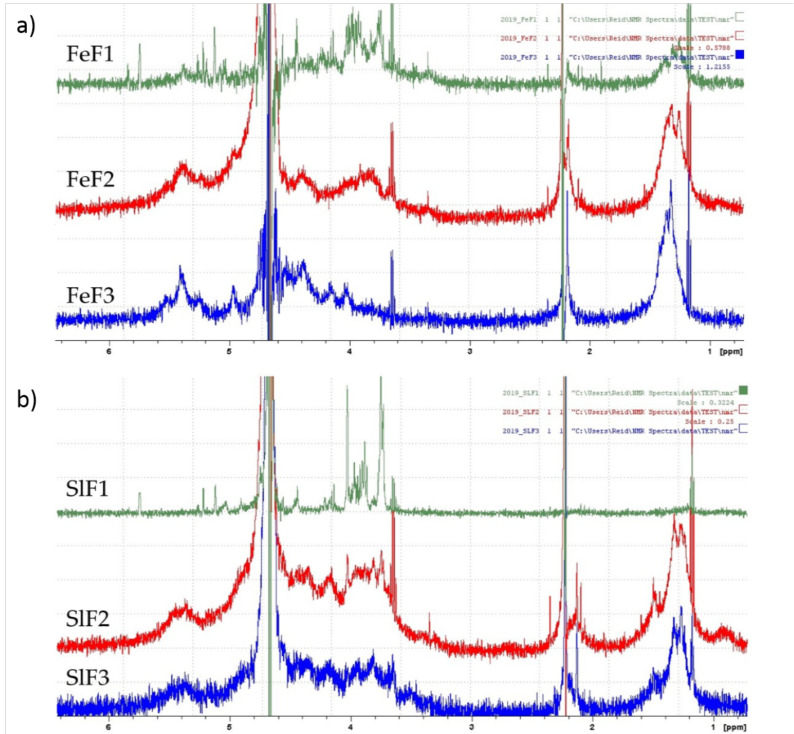
^1^H NMR spectra of fucoidan fractions. (**a**) *F. evanescens* (FeF1, FeF2, FeF3) and (**b**) *S. latissima* (SlF1, SlF2, SlF3) in D_2_O.

^1^H NMR spectroscopy was used for the preliminary determination of fucoidans from *F. evanescens* and *S. latissima*. The spectra displayed several signals that are indicative of the diversity and complexity of the fucoidans. However, several specific signals for fucose were observed in the ^1^H NMR spectra from fucoidan fractions of both seaweeds (Figure 4), such as signals of anomeric protons (5–5.6 ppm), ring protons (3.6–4.8 ppm), and methyl protons (1.2–1.5 ppm) regions [34].

The spectra of all three *F. evanescens* fractions contained specific chemical shifts characteristic of fucoidans (Figure 4a). Only the FeF1 fraction gave signals in the region of 5.8 ppm, characteristic for uronic acids, confirming the presence of alginate impurities that are consistent with the monosaccharide and SEC analysis. Fraction FeF2 and FeF3 were purer than FeF1 and the characteristic peak of uronic acids was not observed. Indications of 1→3 linked l-fucose were detected in the high-field signals at 1.2–1.3 ppm with high intensity. In addition, signals with low intensity at around 1.4 ppm were also observed, which confirmed the presence of 1→4 linked l-fucose, as anticipated [35,36].

The ^1^H NMR spectra of fucoidan fractions from *S. latissima* (Figure 4b) were similar to the spectra of *F. evanescens*, though the methyl proton signal was absent in the SlF1 fraction. The specific resonances of uronic acid at 5.74 ppm (~5.8 ppm) were detected in SlF1, which confirmed the presence of alginate in this fraction. In the fractions SlF2 and SlF3, methyl signals appeared at around 1.3 ppm, with high intensity indicating the 1→3 linked L-fucose in the fucoidan structure, as anticipated [35,36].

## 3. Discussion

The use of a combination of the alginate lyase SALy and the cellulase preparation Cellic^®^CTec2 was effective in extracting fucoidans from both the brown algae, *F. evanescens* and *S. latissima*, which were used here as prototype fucoidan sources to demonstrate the validity of enzyme-assisted extraction. The yields of fucoidans extracted by the enzymatic method were comparable to those that were obtained by mild chemical extraction. The monosaccharide composition of the crude fucoidan extracts differed between the two methods. The chemical extract contained a relatively higher percentage of fucose than the crude enzyme-assisted extract, but that was mainly due to the lower levels of alginate and, as expected, the fucose content in the enzyme-assisted extracts after IEX purification was indeed considerably higher than in the crude chemical extracts. In addition, the galactose content, which is a minor component of fucoidan, was considerably higher in the enzyme-assisted IEX extracts as compared to the chemical extracts. Importantly, the sulfate content was slightly higher in the crude enzyme-assisted extracts than in the chemical extracts. However, the glucose contents of the crude fucoidans that were extracted by the chemical method were higher than the levels obtained by the enzymatic method, and notably much higher in the fucoidan extracted from *S. latissima* as compared to that from *F. evanescens* (Table 2). The analyzed glucose in the fucoidan extracts implies the presence of residual components of laminarin. As mentioned, the difference in glucose levels between the chemical and enzyme-assisted extracts indicates that laminarin, as expected, is readily degraded by Cellic^®^CTec2 in the enzyme-assisted method. The glucose liberated by this treatment is then removed by the ethanol precipitation. This result is in complete agreement with a previous report, where Cellic^®^CTec2 was shown to catalyze the release of glucose and other monosaccharides from *Laminaria digitata* and shown to catalyze the degradation of pure laminarin [31].

The alginate was not fully degraded in the crude extract from the enzyme-assisted purification. The CaCl_2_ precipitation removed higher molecular weight alginates remaining after the enzyme treatment, but low molecular weight alginates were still present in the extracts. These alginates did not precipitate with CaCl_2_ addition, most likely due to their low molecular weight, but presumably also due to the high amount of relative ManA as compared to GuluA, where notably the latter forms gels with CaCl_2_ [37]. The results correlate with the substrate specificity of the alginate lyase used in the extraction process. According to Manns et al [28], the alginate lyase SALy has higher specificity towards poly-(G) than poly-(M) blocks in alginate, consequently leaving behind poly- and oligo-(M) blocks [28]. The alginate lyase was added in excess and it was not an apparent limiting factor, although enzyme inhibitory compounds, like polyphenols, might be present in the seaweed. In the future, the use of bifunctional alginate lyase, or other alginate lyases with higher M-block specificity might improve the purity of the crude fucoidans. Although the low molecular weight alginates were easily removed by IEX, the elimination of this step would be preferable. In literature, many bifunctional recombinant alginate lyases with higher M-block specificity are found, but they all have apparent high pH optima around 8–8.5 [38,39,40,41], which would not work in concert with the Cellic^®^CTec2, which has a pH optimum of 5.0–5.5. Recently, a fungal alginate lyase from *Paradendryphiella salina* with preference for mannuronate was discovered [42], this enzyme and other new alginate lyase enzymes may be developed for large scale use in a protocol in which the IEX procedure could be omitted.

As previously reported, the sulfate content is decreased by acid extraction [43]. According to published data, sulfate plays an important role in the bioactivity of fucoidan, such as anti-angiogenic and antitumor [44], anticoagulant and antiproliferative effects [45], reduction of cancer cell viability *in vitro*, and immunostimulatory effects, such as activation of natural killer cells [46]. The sulfate content was slightly higher in the enzymatically extracted crude fucoidans, even though a gentle method with only 3 h extraction at 70 °C was used for the chemical extraction. An expected higher bioactivity of fucoidan following the enzymatic method on account of higher sulfate content needs further investigation.

The molecular sizes of crude fucoidans and fractions were analyzed in order to evaluate fucoidan degradation during chemical vs. enzyme-assisted extraction. As expected, the molecular weights of crude fucoidans from *F. evanescens* and *S. latissima* were quite high after the enzymatic extraction as compared to the chemical extraction, which is likely related to the extraction conditions. In the chemical procedure, hot acid treatment probably contributed to partial degradation of the fucoidans and resulted in polysaccharides with lower molecular weight. In contrast, in the enzyme-assisted extraction, the selectivity of the enzymes was exploited in order to specifically degrade only the target non-fucoidan polysaccharides in the cell wall of seaweed as well as laminarin, avoiding fucoidan depolymerization and desulfatation.

The molecular weight of crude fucoidans from *F. evanescens* has been previously reported to have various sizes from 181 to 400 kDa after extraction with aqueous CaCl_2_ [47] and HCl purification [48]. In this study, the average size of crude fucoidans from *F. evanescens* was 200–400 kDa, which is a high molecular size when compared with fractions that were extracted by acid (from 150 to 500 kDa) [49] and by ultrasound-assisted extraction (280 and 240 kDa) [50]. Fucoidans from *F. evanescens* were previously reported as a fucansulfate [10]. This interpretation of the *F. evanescens* fucoidans being fucansulfate molecules was confirmed by the high amount of fucose and the monomodal profile of SEC curves of the pure fractions FeF2 and FeF3. Characteristic signals confirmed the presence of fucose and also indicated 1→4 and 1→3 linked L-fucose in the ^1^H NMR spectra of fractions from *F. evanescens* (Figure 4). This is in agreement with the data reported by Bilan et al. [10].

The fucoidans from *S. latissima* were more complex based on their monosaccharide composition. In addition, the SEC profiles for fraction SlF2 and SlF3 showed broad double peaks, suggesting that they might be large heterogeneous polysaccharides [47]. Three types of sulfated fucan-polysaccharides have been reported in *S. latissima*: fucogalactan, fucoglucuronomannan, and fucoglucuronan [13]. The average molecular weights of crude fucoidans and fractions from this seaweed were very high. Recently, fucoidan fractions of *S. latissima* extracted by an aqueous CaCl_2_ method with sizes up to 543 kDa were reported [51]. With the enzyme-assisted extraction method fucoidans with even higher molecular weights were obtained. ^1^H NMR spectra of fucoidan fractions of *S. latissima* confirmed the presence of fucose 1→3 linked L-fucose, similar to published data [10,13].

## 4. Material and Methods

### 4.1. Materials

*S. latissima* from Iceland, harvested in June 2017, was provided as dried flakes by Blaskel (Stykkisholmur, Iceland). *F. evanescens*, from Kiel fjord, Germany, was collected in March 2017 and provided as fresh frozen by Coastal Research & Management (Kiel, Germany). The *F. evanescens* seaweed was washed with fresh water to remove impurities and lyophilized. The dried *F. evanescens* seaweed was ground into powder (~0.5 mm) and stored at room temperature.

Cellic^®^CTec2 was obtained from Novozymes (Bagsværd, Denmark). This is a commercial cellulase preparation based on the *Trichoderma reesei* cellulolytic enzyme complex; this preparation had an activity of 142 FPU/mL (filter paper activity units). Apart from the cellulolytic enzyme base from *T. reesei* RUT C-30 containing at least the two main cellobiohydrolases EC 3.2.1.91 (Cel6A and Cel7A), several different endo-1,4-β-glucanases EC 3.2.1.4 (Cel7B, Cel5A, Cel12A, Cel45A), β-glucosidase EC 3.2.1.21, and a GH3 β-xylosidase EC 3.2.1.37; this preparation includes extra β-glucosidase and lytic cellulose monooxygenases (1.14.99.54, 1.14.99.56, AA9) and other proprietary proteins.

### 4.2. Alginate Lyase Expression and purification

The overnight culture of *E. coli* hosting the *Sphingomonas* sp. alginate lyase SALy was prepared, as described in Manns et al. 2016 [28]. The expression was performed in 5 L fermenters in auto-induction media (6 g Na_2_HPO_4_, 3 g KH_2_PO_4_, 20 g tryptone, 5 g yeast extract, 5 g NaCl, 0.06% v/v glycerol, 0.05% w/v glucose, and 0.04% w/v α-lactose, pH 7.2) at 20 °C. The cells were collected by centrifugation, re-suspended in cold extraction-buffer (20 mM Tris-HCl buffer, pH 7.4, 250 mM NaCl, 2 mg/mL lysozyme), sonicated to destroy the cell wall, and then centrifuged to remove debris. The supernatant was collected and passed through a 0.22 µm filter, and then loaded onto a Ni^2+^-Sepharose HisTrap HP column (GE Healthcare, Uppsala, Sweden). Unbound material was washed off the column with 10 column volumes of wash buffer 20 mM Tris-HCl, pH 7.4, 250 mM NaCl, 20 mM imidazole. The enzyme was eluted with elution buffer (20 mM Tris-HCl, pH 7.4, 250 mM NaCl, 100 mM imidazole). The enzyme activity of the alginate lyase was ~18 units/mg enzyme quantified as %substrate consumption/(min·mg enzyme) calculated from formation of double bonds at 235 nm on sodium alginate at 40 °C, pH 7 [28]. SDS-PAGE confirmed protein purity and the protein concentration was determined by the Bradford method [52].

### 4.3. Fucoidan Extraction from Brown Seaweeds

#### 4.3.1. Chemical Extraction Method

**Figure 5:**
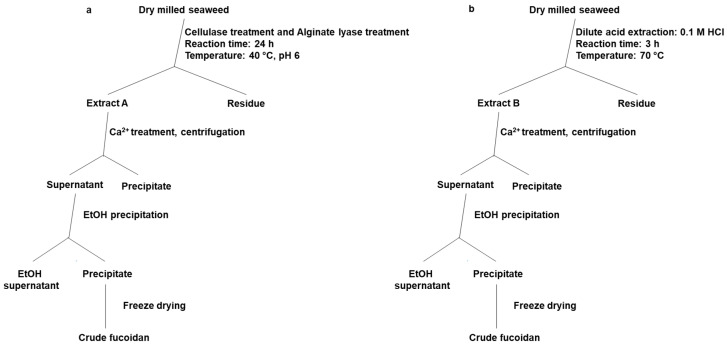
Flow-chart of extraction procedures. (**a**) Enzyme-assisted extraction and (**b**) Chemical extraction (mild acid extraction). (EtOH is ethanol).

The chemical extraction of crude fucoidans was performed in 0.1 M HCl solution with a ratio of seaweed to extracting liquid of 1:20 and treatment for 3 h at 70 °C (methodology modified from Ale et al. 2012 [53]). The supernatant (Extract A, Figure 5a)) was collected by centrifugation for 10 min. at 19000 rpm. Next, 2% CaCl_2_ solution was added to remove alginate as a precipitate after centrifugation (Figure 5). The crude fucoidans were isolated from the supernatant by 72% ethanol (EtOH), recovered by centrifugation at 19000 rpm for 30 min, and lyophilized. The latter steps were comparable to the enzyme-assisted extraction method (Figure 5).

#### 4.3.2. Enzyme-Assisted Extraction Method

The enzymatic extraction of fucoidans was initiated by a combined cellulase and alginate treatment (Figure 5): in practice, the first step included simultaneous addition of the enzymes at a level of 5% (v/w) for Cellic^®^CTec2, and 0.35% (w/w) for alginate lyase. The treatment was conducted at 40 °C for 24 h on a horizontal mixer at 100 rpm performed in 55 mM phosphate-38 mM citrate buffer pH 6 with 5% (w/v) substrate concentration. The reaction was stopped by boiling at 90 °C for 10 min and then cooling on ice. The supernatant (Extract B, Figure 5b) was collected after centrifugation for 10 min at 19000 rpm. To remove residual alginate, 2% CaCl_2_ was added and the mixture was centrifuged again. The fucoidans were precipitated from the supernatant after alginate removal and isolated by the addition of EtOH to a final concentration of 72%, recovered by centrifugation at 19000 rpm for 30 min, and lyophilized.

### 4.4. Fucoidan Fractionation by Anion-Exchange Chromatography

Crude fucoidan samples in aqueous solution (5 g in 100 mL) were applied to a column (2.6 cm × 40 cm) that had been manually packed with DEAE-Macroprep resin material (Bio-Rad, CA, USA) and then equilibrated with acidic NaCl (0.04 N HCl in 0.1 M NaCl) (packing was done according to the instruction manual (Lit271 Rev D) from Bio-Rad). The unbound materials were washed from the column with 0.1 M NaCl. Fucoidans were eluted at a flow rate of 5 mL/min in a sequential concentration gradient of NaCl from 0.1 to 2M. The eluted fractions were combined based on the results of total carbohydrate analysis (reducing ends analysis) by the phenol-sulfuric acid method [32]. The fractions were passed through a 10 kDa membrane in order to concentrate the fucoidan and remove salt and then lyophilized.

### 4.5. Chemical Composition Analysis

#### 4.5.1. Two-Step Acid Hydrolysis

Seaweed and fucoidan samples (5 mg) were hydrolyzed in 50 µL 72% H_2_SO_4_ at 30 °C for 1 h and the mixture was then diluted to 4% H_2_SO_4_ and hydrolysis continued for an additional 40 min at 120 °C in an autoclave. The hydrolysates were neutralized, filtered through a 0.22 µm syringe filter, and used for monosaccharide analysis [54].

#### 4.5.2. Chemical Composition Analysis

Monosaccharide composition, mannitol, and uronic acids of the hydrolysates were analyzed on a Dionex ICS-3000 HPAEC-PAD system with pulsed amperometric detection (PAD). Three eluents were used: A—deionized water, B—200 mM NaOH, and C—200 mM NaOH, 1 M NaOAc. Chromatographic separation was performed with a flow rate 0.4 mL/min, using 0.5% B in A for the first 17 min for elution of neutral sugars and sugar alcohol. Next, 3% B and 6% C in A were applied for 20 min for the separation of uronic acid. The process was completed with 100% B in 6 min, after which 0.5% B in A was applied to calibrate the column. Data quantification was analyzed by Chromeleon™ 7.2 (Thermo Scientific). Recovery values of the monosaccharides and uronic acid were estimated from runs at the same time [54].

#### 4.5.3. TFA Hydrolysis and Sulfate Content Analysis

Fucoidan samples (5–6 mg) were hydrolyzed in 1 mL 2 M trifluoroacetic acid (TFA) at 100 °C for six hours (in closed vials in a thermostatted water bath). After hydrolysis, the TFA was evaporated and the residual TFA was removed by the addition of 2.5% ammonium hydroxide (NH_4_OH). The hydrolysates were used to determine the sulfate content by the barium chloride (BaCl_2_) gelatin method [55]. 0.5% gelatin solution was prepared in warm water (60–70 °C). 0.5 % BaCl_2_ was dissolved in gelatin solution and then allowed to stand for 2 h at 25 °C, then centrifuged at 10,000× g for 10 min. 10 μL of hydrolysate solution was added to 160 µL trichloroacetic acid (TCA) and 100 µL BaCl_2_-gelatin reagent. The mixture was allowed to stand for 30 min. A blank was prepared with 170 µl TCA and 100 µL BaCl_2_-gelatin reagent. The released BaSO_4_ suspension was measured at λ = 360 nm in a microplate reader (TECAN Infinite 200, Salzburg, Austria) while using UV-transparent 96-well microplates (Corning^®^, Tewksbury, MA, USA). Potassium sulfate was used as standard to generate a (linear) standard curve for the sulfate response at 360 nm.

### 4.6. Determination of Molecular Weight by Size Exclusion Chromatography

The molecular weights (MW) of fucoidan fractions were determined by High Performance Size Exclusion Chromatography (HP-SEC) using an Ultimate iso-3100SD pump with WPS-3000 sampler (Dionex, Sunnyvale, CA, USA) connected to an RI-101 refractive index detector (Shodex, Showa Denko K.K., Tokyo, Japan). The samples were dissolved in 1 ml HP-SEC buffer (100 mM sodium acetate, pH 6) and filtered through 0.22 μm filters. 100 µL of fucoidan samples (3 mg/mL) were injected into a Shodex SB-806 HQ GPC column (300 × 8 mm) coupled with a Shodex SB-G guard column (50 mm × 6 mm) (Showa Denko K.K., Tokyo, Japan) [56]. Elution was carried out at a flow rate of 0.5 mL/min at 40 °C [56]. Pullulan samples of molecular weight 1, 5, 12, 110, 400, and 800 kDa were used as standards [56].

### 4.7. ^1^H NMR Analysis

NMR spectra were recorded using an Avance III-700 NMR spectrometer (BrukerBiospin AG, Switzerland) and Avance III-500 HD NMR spectrometer (Bruker, Germany). The samples (3 mg) were prepared by dissolving freeze-dried fucoidan fractions in 550 µl of deuterated water (D_2_O) and then placing them in 5 mm tubes. The ^1^Н spectra were recorded at 35 °C with acetone as the internal standard (2.225 ppm).

## 5. Conclusions

An enzyme-assisted extraction method for fucoidans from brown seaweeds was introduced and compared to mild chemical extraction of fucoidan from *F. evanescens* and *S. latissima*. The enzyme-assisted procedure, including IEX purification, resulted in highly satisfactory yields of pure fucoidans having similar or slightly higher sulfate, and a higher molecular weight than the fucoidans obtained with the chemical acidic extraction method. Therefore, the enzyme-assisted extraction method appears to be promising for obtaining authentic brown seaweed fucoidans of high bioactivity and, in particular, offers a new approach that can continue to be improved to further advance the understanding of the structure-bioactivity effects of fucoidans and promote further sustainable explorations of fucoidans.

## Figures and Tables

**Table 1:** Monosaccharide composition of the brown seaweeds. The data are given as % weight (dehydrated monomers) of dry matter. Different superscript roman letters a,b indicate statistically different values (*p* < 0.05) pairwise between values from each type of seaweed.

Monomer Category	Monomer	*F. evanescens*	*S. latissima*
Neutral monosaccharides (%)	Mannitol	2.6 ^a^ ± 0.6	2.1 ^a^ ± 0.5
	Fucose	8.7 ^a^ ± 0.9	4.7 ^b^ ± 0.1
	Rhamnose	0.1 ^a^ ± 0.0	0.1 ^a^ ± 0.0
	Galactose	1.5 ^a^ ± 0.4	0.5 ^b^ ± 0.5
	Glucose	6.7 ^b^ ± 0.8	12.3 ^a^ ± 2.4
	Xylose	0.8 ^a^ ± 0.0	0.4 ^b^ ± 0.1
	Mannose	0.9 ^a^ ± 0.2	1.0 ^a^ ± 0.3
Uronic acids (%)	Guluronic Acid	8.8 ^a^ ± 1.1	9.3 ^a^ ± 2.4
	Glucuronic Acid	1.2 ^a^ ± 0.2	1.9 ^a^ ± 0.5
	Mannuronic Acid	28.3 ^a^ ± 3.6	36.1 ^a^ ± 8.8

**Table 2:** Chemical composition of crude fucoidans from *F. evanescens* and *S. latissima.* The monosaccharide and uronide data are given in %mol (relative level) of total carbohydrates analysed in the extract, with total sulfate (SO_4_^2−^) first calculated as %wt of total, then as degree of sulfation on dehydrated fucose moieties in the crude fucoidans extracted. Fucoidan yield is calculated as amount of fucose extracted compared to the total fucose (theoretical maximum) in the starting-material. Different superscript roman letters a,b indicate statistically different values (*p* < 0.05) pairwise for the enzymatic method vs. the chemical method per seaweed species.

Content	Monomer	*F. evanescens*		*S. latissima*	
		Enzymatic Method	Chemical Method	Enzymatic Method	Chemical Method
Neutral monosaccharides (%mol)	Mannitol	0.2 ^b^ ± 0.0	0.43 ^a^± 0.0	2.1 ^a^ ± 0.2	2.2 ^a^ ± 0.3
	Fucose	24.8 ^b^ ± 2.9	60.9 ^a^ ± 0.9	12.6 ^b^ ± 0.4	31.2 ^a^ ± 4.2
	Rhamnose	0.2 ^b^ ± 0.1	0.9 ^a^ ± 0.2	0.2 ^a^ ± 0.0	0.2 ^a^ ± 0.0
	Galactose	0.9 ^b^± 0.1	5.4 ^a^ ± 0.1	2.3 ^a^ ± 0.1	2.9 ^a^ ± 2.4
	Glucose	0.7 ^b^ ± 0.1	6.2 ^a^ ± 0.1	1.6 ^b^ ± 0.0	57.7 ^a^ ± 3.1
	Xylose	0.8 ^b^ ± 0.1	5.8 ^a^ ± 0.1	0.8 ^b^ ± 0.0	3.0 ^a^ ± 0.0
	Mannose	0.4 ^b^ ± 0.0	2.6 ^a^ ± 0.1	0.9 ^a^ ± 0.0	0.9 ^a^ ± 0.1
Uronic acid (%mol)	GuluA	12.6 ^a^ ± 1.8	0.9 ^b^ ± 0.1	18.6 ^a^ ± 0.9	0.2 ^b^ ± 0.1
	GluA	1.0 ^b^ ± 0.2	3.9 ^a^ ± 0.1	1.3 ^a^ ± 0.2	0.7 ^b^ ± 0.1
	ManA	58.4 ^a^ ± 2.6	13.1 ^b^ ± 0.4	59.6 ^a^ ± 1.9	1.0 ^b^ ± 0.2
Sulfate (SO_4_^2−^) (%wt)		21.4 ^b^ ± 0.5	38.0 ^a^ ± 0.4	15.5 ^b^ ± 1.7	31.6 ^a^ ± 0.9
Degree of sulfation (molar ratio SO_4_^2−^: Fucose)		2.1	1.9	2.5	2.1
Fucoidan yield (fucose extraction %wt)		40	43	29	29

**Table 3:** Yields and composition of fucoidan fractions from *F. evanescens* and *S. latissima.* Different superscript roman letters a,b,c indicate statistically different values (*p* < 0.05) between the values in the fractions per seaweed species.

Content	Monomer	*F. evanescens*			*S. latissima*		
		FeF1	FeF2	FeF3	SlF1	SlF2	SlF3
Yield, % of crude extract		4.2	7.9	18.2	3.4	6.2	3.8
Neutral monosaccharides (%mol)	Mannitol	0.0 ^a^ ± 0.0	0.0 ^a^ ± 0.0	0.0 ^a^ ± 0.0	0.0 ^a^ ± 0.0	0.1 ^a^ ± 0.0	0.0 ^a^ ± 0.0
	Fucose	34 ^c^ ± 3.1	74.7 ^b^ ± 0.8	87.8 ^a^ ± 1.4	5.4 ^b^ ± 1.2	64.7 ^a^ ± 0.3	63.3 ^a^ ± 0.7
	Rhamnose	0.3 ^c^ ± 0.1	0.8 ^a^ ± 0.1	0.5 ^b^ ± 0.1	0.1 ^b^ ± 0.0	0.3 ^a^ ± 0.0	0.3 ^a^ ± 0.0
	Galactose	4.6 ^c^ ± 0.4	15.4 ^a^ ± 0.4	9.0 ^b^ ± 0.9	0.5 ^c^ ± 0.0	12.2 ^b^ ± 0.1	26.9 ^a^ ± 0.3
	Glucose	7.7 ^a^ ± 0.7	1.4 ^b^ ± 0.1	0.3 ^c^ ± 0.1	0.4 ^b^ ± 0.0	0.6 ^a^ ± 0.1	0.4 ^b^ ± 0.1
	Xylose	5.3 ^a^ ± 0.5	2.8 ^b^ ± 0.1	1.5 ^c^ ± 0.3	0.8 ^c^ ± 0.1	4.8 ^a^ ± 0.0	3.4 ^b^ ± 0.2
	Mannose	3.2 ^a^ ± 0.5	2.3 ^b^ ± 0.1	0.3 ^c^ ± 0.1	0.8 ^c^ ± 0.1	3.5 ^a^ ± 0.2	2.1 ^b^ ± 0.1
Uronic acid (%mol)	GuluA	9.1 ^a^ ± 0.5	2.2 ^b^ ± 0.2	0.0 ^c^ ± 0.0	1.1 ^c^ ± 0.1	6.9 ^a^ ± 0.3	2.8 ^b^ ± 0.2
	GluA	3.8 ^a^ ± 0.3	0.3 ^b^ ± 0.0	0.5 ^b^ ± 0.1	8.5 ^a^ ± 4.7	0.0 ^b^ ± 0.0	0.0 ^b^ ± 0.0
	ManA	32.2 ^a^ ± 0.6	0.2 ^b^ ± 0.0	0.0 ^b^ ± 0.0	82.4 ^a^ ± 4.3	6.9 ^b^ ± 0.1	0.8 ^c^ ± 0.1
Sulfate (SO_4_^2−^) (wt%)		20.4 ^b^ ± 3.4	34.8 ^ab^ ± 2.0	38.7 ^a^ ± 1.0	6.6 ^c^ ± 3.6	35.6 ^b^ ± 2.5	46.4 ^a^ ± 3.5
Degree of sulfation (molar ratio SO_4_^2−^: Fuc)		1.3	1.7	1.6	1.8	2.4	3.0
